# Survival analysis of palliative radiotherapy in patients with HER-2+ metastatic breast cancer

**DOI:** 10.3389/fendo.2023.1305429

**Published:** 2024-01-08

**Authors:** Xueting Li, Xiaorong Zhong, Hongyu Xu, Jun Wang, Xianguo Liu, Yang Wang, Liang He, Jiayu Ma, Guanghua Li, Lei Liu

**Affiliations:** ^1^ Division of Head & Neck Tumor Multimodality Treatment, Cancer Center, West China Hospital, Sichuan University, Chengdu, Sichuan, China; ^2^ Department of Oncology, 363 Hospital, Chengdu, Sichuan, China; ^3^ Department of Breast Center, West China Hospital, Sichuan University, Chengdu, Sichuan, China

**Keywords:** radiotherapy, HER-2, breast cancer, overall survival, targeted therapy for metastatic breast cancer

## Abstract

**Background:**

Whether radiotherapy can improve the long-term survival of HER-2+ metastatic breast cancer remains unclear. We launched this study to explore the effect of HER-2+ metastatic breast cancer patients through anti-HER-2 targeted therapy + radiotherapy.

**Methods:**

488 HER-2 + metastatic breast cancer patients who received anti-HER2 targeted ± local radiotherapy from March 2006 to September 2021 were retrospectively collected. Patients were divided into a radiotherapy group (n=207) and a non-radiotherapy group (n=281) based on whether they received radiotherapy or not. 1: 1 propensity matching analysis was used to determine two groups of patients with similar baselines.

**Results:**

Before matching, the radiotherapy group (n=207) had a median overall survival (mOS) of 51.7 months (48.8-63.8), which was superior to the non-radiotherapy group’s (n=281) mOS of 33.9 months (27.9-39.9) (P < 0.0001). Moreover, the radiotherapy group exhibited better 1-year (94.6% vs 83.9%), 3-year (70.8% vs 45.5%), and 5-year (43.3% vs 25.0%) survival rates compared to the control group. Propensity score matching analysis identified 135 pairs of baseline-matched patients. In the matched groups, the mOS was 57.2 (44.5-69.8) months in the radiotherapy group (n=135) and 34.1 (27.5-40.6) months in the non-radiotherapy group (n=135), showing a statistically significant difference (P < 0.0001). Additionally, the radiotherapy group demonstrated 1-, 3-, and 5-year survival rates of 93.2%, 71.5%, and 46.9%, respectively, while those in the non-radiotherapy group were 89.4%, 45.8%, and 22.2%, respectively. Multivariate Cox analysis revealed that the presence of brain metastasis, liver metastasis, and radiotherapy were identified as independent predictive factors significantly associated with OS.

**Conclusion:**

In patients with HER-2 positive metastatic breast cancer, radiotherapy was associated with better survival benefits compared to those who did not receive radiotherapy.

## 1 Introduction

In 2021, breast cancer ranked first among female cancer incidence rates and was the fourth leading cause of cancer-related deaths in Chinese women (1). In 1989, the mortality rate of breast cancer in American women reached its highest point and gradually decreased thereafter (2). This is closely related to early detection, timely effective treatment, and the development of treatment techniques. However, about 20-30% of cases are diagnosed as metastatic breast cancer (3). The goal of treatment is to improve the quality of life and extend survival. Currently, systemic therapy is the primary approach, with local therapy as a complement. As a heterogeneous disease, the survival outcomes for metastatic breast cancer vary widely, with a 2-year progression-free survival (PFS) of approximately 10% to 40% and a median overall survival (mOS) of approximately 10 to 38 months (4, 5).

Statistics indicate that approximately 20% of breast cancer patients exhibit overexpression of human epidermal growth factor receptor 2 (HER2), which is associated with adverse prognosis, an aggressive phenotype, and shorter OS (6). This receptor is located on chromosome 17q21 and, when activated through dimerization or cleavage of its extracellular domains, triggers signal cascades in the MAP kinase pathway or PI3K/Akt/MTOR system, leading to cell proliferation and promoting the division and growth of cancer cells (7). Currently, trastuzumab and pertuzumab are the most commonly used drugs in HER2-targeted therapy (8). Trastuzumab, as the first humanized monoclonal antibody targeting HER2 receptors, primarily acts on the extracellular domains of the receptor, blocking the MAP kinase signaling pathway and the PI3K-Akt pathway, thereby slowing the cell cycle and reducing tumor cell proliferation (9). This drug can also recruit innate immune cells, macrophages, and NK cells (10). Its efficacy in HER2-positive breast cancer has been confirmed at various disease stages (11).

Radiation therapy (RT) holds a significant position in the treatment of breast cancer. In advanced metastatic breast cancer, local RT primarily aims to provide palliative care, alleviate local symptoms, and improve local control. For breast cancer patients with bone metastasis, it can alleviate pain and reduce the incidence of pathological fractures and other bone-related events. For patients with brain metastases, individualized choices of whole-brain RT (WBRT) and stereotactic body RT (SBRT) can improve the mOS of brain metastasis patients. With the development of precision RT techniques, it can also serve as a local treatment modality for breast cancer in other sites such as the liver and lungs, enhancing local control. Multiple studies have shown that radiation therapy can improve the prognosis of stage IV breast cancer patients after treatment (12, 13). A prospective study involving 581 metastatic breast cancer patients demonstrated a 30% reduction in the risk of death after receiving RT (14). Steinauer K et al. (15) also reported that radiation therapy could extend the mOS of metastatic breast cancer patients by approximately 12 months.

However, some researchers still believe that RT has limited long-term control of recurrent or metastatic breast cancer and provides minimal benefit to advanced breast cancer patients. Vlastos et al. (16) conducted a study that did not yield conclusive results regarding the impact of radiation therapy on the prognosis of late-stage breast cancer patients, indicating that the debate about whether RT provides survival benefits for late-stage breast cancer patients remains unresolved. Adverse events associated with radiation therapy, such as bone marrow suppression, gastrointestinal toxicity, pulmonary toxicity, and cardiac toxicity, are also relatively common, and the benefit of radiation therapy for late-stage metastatic breast cancer patients still requires further confirmation.

While most current data suggest that the toxicity of RT plus targeted therapy is tolerable, data on the effectiveness and toxicity of this combination therapy remain very limited. Therefore, we have initiated this study to explore the effectiveness of treatment for HER2-positive metastatic breast cancer patients through anti-HER2 targeted therapy combined with or without local RT.

## 2 Materials and methods

### 2.1 Patients

The patient cohort was sourced from the case databases of three tertiary grade-A hospitals in China, including Sichuan University West China Hospital. Data was retrospectively collected from HER-2 positive metastatic breast cancer patients who received anti-HER2 targeted therapy ± local radiation therapy between March 2006 and September 2021. Patients were categorized into two groups (RT group and Non-RT Group) based on whether they underwent local RT.

Inclusion criteria: 1) pathologically confirmed malignant tumors originating from the breast; 2) stage IV (AJCC 8th edition) metastatic breast cancer patients; 3) HER-2 positive results (HER-2+++, if HER-2++, further confirmation of HER-2 amplification status was determined by FISH testing); 4) received anti-HER2 targeted therapy, which may be combined with chemotherapy; 5) presence of measurable lesions.

Exclusion criteria: 1) Age <18 or >80 years; 2) non-female patients; 3) incomplete or lost follow-up information; 4) predicted survival of less than 3 months; 5) presence of severe illnesses that would interfere with planned treatment, especially severe heart disease, cerebrovascular disease, or severe lung disease.

This study has received approval from the Ethics Committee of West China Hospital. Due to the retrospective nature of this research, informed consent was waived.

### 2.2 Treatment protocol

#### 2.2.1 RT

The RT modalities include intensity-modulated RT (IMRT), SBRT, and three-dimensional conformal RT (3D-CRT). RT is administered using linear accelerators or cobalt-60 therapy machines. The target area and dosage are outlined and designed based on RTOG recommendations, depending on the irradiation site. For example, common radiation doses for bone metastases include: 30 Gy/10 fractions, 20 Gy/5 fractions, or 8 Gy/single fraction; while whole-brain radiation therapy (WBRT) doses are typically 30 Gy/10 fractions or 40 Gy/20 fractions.

#### 2.2.2 Molecular targeted therapy

The usage and dosage of molecular targeted therapies are recommended according to authoritative guidelines such as NCCN and CSCO. For example: Trastuzumab is administered at a loading dose of 8 mg/kg followed by 6 mg/kg every 21 days; Pertuzumab is given at a loading dose of 840 mg followed by 420 mg every 21 days; Lapatinib is taken orally at 400 mg once daily; Palbociclib is taken orally at 1250 mg once daily; T-DM1 is administered at a dose of 3.6 mg/kg per cycle every 21 days.

#### 2.2.3 Combination chemotherapy

Chemotherapy regimens are recommended in accordance with authoritative guidelines such as NCCN and CSCO, including commonly used chemotherapy protocols in clinical practice, such as anthracycline-based and taxane-based drugs, with the chemotherapy cycle determined based on the specific regimen and individual circumstances.

#### 2.2.4 Follow-up

The primary observation parameter in this study is OS. OS is defined as the time from the start of treatment for advanced metastatic breast cancer until death from any cause or the last follow-up time.

#### 2.2.5 Statistical analysis

We used SPSS 26.0 for statistical analysis. Inter-group comparisons were conducted using the chi-square test and Mann-Whitney U test. 1:1 propensity score matching (PSM) was performed to identify baseline-matched patients in the RT group and non-RT group. Matching variables included age, pathological type, HR expression, Ki-67, T stage, N stage, metastatic sites, chemotherapy, hormone therapy, and targeted therapy. Subsequently, Kaplan-Meier survival analysis and the log-rank test were employed to compare the OS of the two groups before and after matching. Univariate and multivariate Cox analysis was used to identify predictive factors for OS. P<0.05 was considered statistically significant.

## 3 Results

### 3.1 Patient characteristics

In this retrospective study, a total of 488 HER-2 positive metastatic breast cancer patients were included (207 in the RT group and 281 in the non-RT group). Prior to matching, significant differences were observed between the two groups in terms of bone metastasis (P<0.001), brain metastasis (P<0.001), lung metastasis (P<0.001), and chemotherapy regimens (P=0.021). However, after matching, there were no significant baseline differences between the two groups (**Table 1**).

**Table 1:** Baseline characteristics of the patients before and after PSM.

Variable	Before PSM			After PSM		
	RT	Non-RT	P	RT	Non-RT	p
Patients	207	281		135	135	
Age (years)			0.504			0.902
<50	108 (52.2)	138 (49.1)		75 (55.6)	73 (54.1)	
≥50	99 (47.8)	143 (50.9)		60 (44.4)	62 (45.9)	
Pathological Type			0.597			0.117
Ductal carcinoma	193 (93.2)	255 (90.7)		127 (94.1)	124 (91.9)	
Lobular carcinoma	7 (3.4)	14 (5.0)		2 (1.5)	9 (6.7)	
Other	7 (3.4)	12 (4.3)		6 (4.4)	2 (1.5)	
HR Expression			0.700			0.268
Negative	68 (32.9)	97 (34.5)		37 (27.4)	47 (34.8)	
Positive	139 (67.1)	184 (65.5)		98 (72.6)	88 (65.2)	
Ki-67			0.241			1.000
<14	30 (14.5)	52 (18.5)		26 (19.3)	25 (18.5)	
≥14	177 (85.5)	229 (81.5)		109 (80.7)	110 (81.5)	
T Stage			0.228			0.640
T1	11 (5.3)	29 (10.3)		8 (5.9)	11 (8.1)	
T2	88 (42.5)	116 (41.3)		55 (40.7)	48 (35.6)	
T3	57 (27.5)	67 (23.8)		42 (31.1)	47 (34.8)	
T4	51 (24.6)	69 (24.6)		30 (22.2)	29 (21.5)	
N Stage			0.261			0.742
N0	21 (10.1)	36 (12.8)		14 (10.4)	10 (7.4)	
N1	55 (26.6)	92 (32.7)		40 (29.6)	46 (34.1)	
N2	61 (29.5)	75 (26.7)		41 (30.4)	37 (27.4)	
N3	70 (33.8)	78 (27.8)		40 (29.6)	42 (31.1)	
Metastatic Sites						
Bone	127 (61.4)	115 (40.9)	<0.001	69 (51.1)	67 (49.6)	0.906
Brain	92 (44.4)	49 (17.4)	<0.001	43 (31.9)	40 (29.6)	0.795
Liver	90 (43.5)	126 (44.8)	0.765	62 (45.9)	55 (40.7)	0.450
Lung	124 (59.9)	124 (44.1)	0.001	73 (54.1)	80 (59.3)	0.435
Other	63 (30.4)	81 (28.8)	0.700	34 (25.2)	34 (25.2)	1.000
Chemotherapy			0.021			0.373
Taxane	124 (59.9)	198 (70.5)		84 (62.2)	90 (66.7)	
Anthracycline	9 (4.3)	6 (2.1)		9 (6.7)	3 (2.2)	
Pyrimidine analog	60 (29.0)	70 (24.9)		40 (29.6)	37 (27.4)	
Other	14 (6.8)	7 (2.5)		2 (1.5)	5 (3.7)	
Hormone Therapy			0.129			0.818
Negative	108 (52.2)	166 (59.1)		70 (51.9)	67 (49.6)	
Positive	99 (47.8)	115 (40.9)		65 (48.1)	68 (50.4)	
Targeted Therapy			0.161			0.128
Trastuzumab	125 (60.4)	192 (68.3)		85 (63.0)	93 (68.9)	
Trastuzumab + pertuzumab	34 (16.4)	33 (11.7)		13 (9.6)	13 (9.6)	
Other	48 (23.2)	56 (19.9)		37 (27.4)	29 (21.5)	

PSM, propensity score matching; RT, radiation therapy.

### 3.2 OS

**Figure 1:**
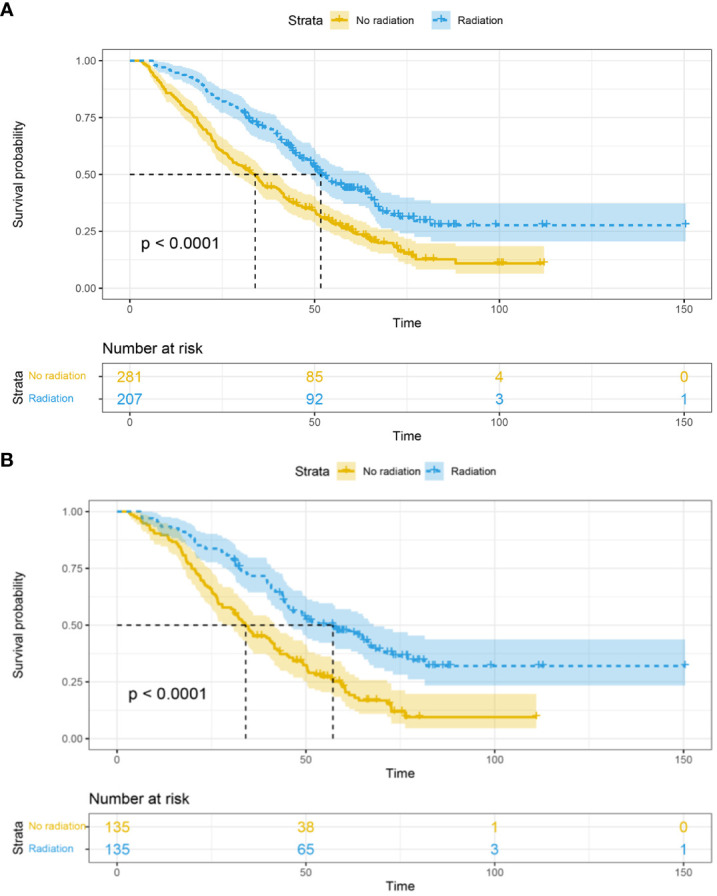
Overall survival in the RT and non-RT groups before **(A)** and after **(B)** propensity score matching. RT, radiation therapy.

Before matching, among the 488 HER-2 positive metastatic breast cancer patients, a total of 344 (70.5%) patients died, with a median follow-up time of 64.9 (62.9-67.0) months. The mOS in the RT group was 51.7 (48.8-63.8) months, which was significantly better than the non-RT group’s mOS of 33.9 (27.9-39.9) months (P<0.0001). Furthermore, the 1-year, 3-year, and 5-year survival rates in the RT group were 94.6%, 70.8%, and 43.3%, respectively, compared to the non-RT group’s rates of 83.9%, 45.5%, and 25.0% (**Figure 1A**).

After matching, among the 270 baseline-matched HER-2 positive metastatic breast cancer patients, a total of 188 (69.6%) patients died, with a median follow-up time of 72.1 (67.7-76.6) months. The mOS in the radiation therapy group (n=135) was 57.2 (44.5-69.8) months, which was significantly better than the non-RT group (n=135) with an mOS of 34.1 (27.5-40.6) months (P<0.0001). Additionally, the 1-year, 3-year, and 5-year survival rates in the RT group were 93.2%, 71.5%, and 46.9%, respectively, compared to the non-RT group’s rates of 89.4%, 45.8%, and 22.2% (**Figure 1B**).

### 3.3 Factors associated with OS

To investigate the risk factors affecting the OS of HER-2 positive metastatic breast cancer patients after matching, we conducted a Cox analysis. The results showed that in the univariate analysis, brain metastasis (HR 1.647, 95% CI 1.217–2.229, P=0.001) and RT (HR 0.361, 95% CI 0.361–0.650, P<0.001) were predictive factors associated with OS in HER-2 positive metastatic breast cancer patients. In the multivariate analysis, both brain metastasis and radiation therapy were identified as independent prognostic factors influencing OS (**Table 2**).

**Table 2:** Univariate and multivariate Cox regression analysis of overall survival after PSM.

Variable	Univariable Cox regression			Multivariable Cox regression		
	HR	95%CI	P	HR	95%CI	P
Age (≥60/<60)	1.034	0.776–1.378	0.820			
HR (positive/negative)	1.031	0.755–1.409	0.846			
Ki-67 (≥14/<14)	0.910	0.642–1.291	0.598			
T stage			0.196			
T1	1.000					
T2	0.640	0.366–1.119	0.117			
T3	0.675	0.383–1.190	0.174			
T4	0.886	0.492–1.594	0.686			
Lymph node metastasis (yes/no)	0.851	0.535–1.354	0.497			
Bone metastasis (yes/no)	0.858	0.644–1.143	0.295			
Brain metastasis (yes/no)	1.647	1.217–2.229	0.001	1.732	1.280–2.343	<0.001
Liver metastasis (yes/no)	1.280	0.961–1.705	0.092			
Lung metastasis (yes/no)	1.146	0.858–1.532	0.356			
RT(yes/no)	0.485	0.361–0.650	<0.001	0.470	0.351–0.631	<0.001

PSM, propensity score matching; RT, External beam radiation therapy.

### 3.4 Subgroup analysis

**Figure 2:**
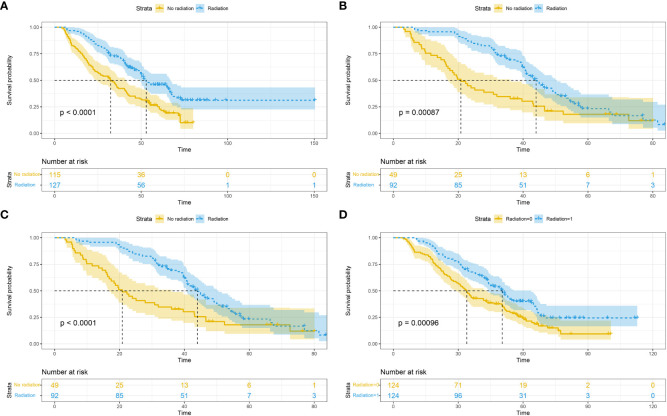
Overall survival based on different metastatic sites: bone **(A)**; brain **(B)**; liver **(C)**; lung **(D)**.

In subgroup analyses combining bone metastasis (52.9 vs. 32.3 months, p<0.0001; **Figure 2A**), brain metastasis (44.0 vs. 20.8 months, p=0.00087; **Figure 2B**), liver metastasis (54.7 vs. 31.7 months, p<0.0001; **Figure 2C**), and lung metastasis (50.4 vs. 34.0 months, p<0.0001; **Figure 2D**), the RT group demonstrated superior median overall survival (mOS) compared to the non-RT group.

**Figure 3:**
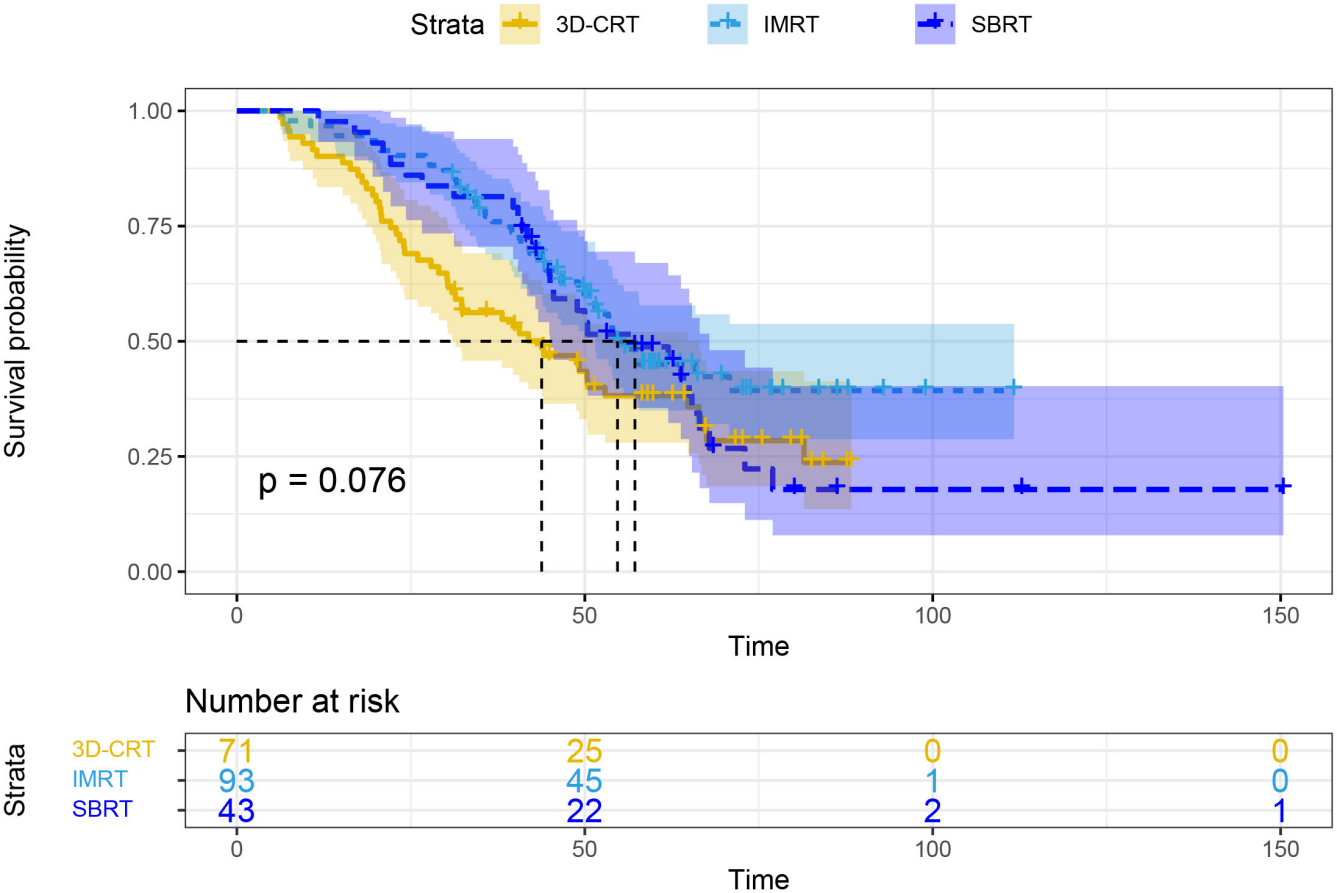
Overall survival based on different radiotherapy modalities. 3D-CRT, three-dimensional conformal radiotherapy; IMRT, intensity-modulated radiotherapy; SBRT, stereotactic body radiotherapy.

In subgroup analyses based on different radiotherapy modalities, the mOS was 43.8 months for the 3D-CRT group, 54.7 months for the IMRT group, and 57.2 months for the SBRT group, with no statistically significant differences (P=0.076; **Figure 3**).

## 4 Discussion

Metastatic breast cancer, whether present at the initial tumor diagnosis or arising as distant progression, is a heterogeneous disease. Its survival rates may be influenced by the tumor characteristics of patients and effective comprehensive treatments (17, 18). In this study, we compared the outcomes of HER2-positive metastatic breast cancer patients receiving anti-HER2 targeted therapy with or without concurrent local radiotherapy. Our findings revealed that the mOS for the RT group was 51.7 (48.8-63.8) months, which was superior to the non-RT group with an mOS of 33.9 (27.9-39.9) months before matching (P < 0.0001). After PSM, RT group demonstrated more pronounced survival benefits (57.2 vs. 34.1 months, P<0.0001) compared to non-RT group. Therefore, we conclude that local palliative radiotherapy can further improve patient survival.

This significant outcome might be attributed to the high local control achieved by radiotherapy (19–23). Studies have shown that breast cancer patients with concurrent bone and brain metastasis often suffer from severe symptoms such as pain, intracranial pressure, pathological fractures, etc. Radiotherapy is not only an effective treatment for improving local symptoms, relieving pain, and reducing the incidence of pathological fractures (24, 25), but also provides a possibility for the application of other anti-tumor measures (26, 27). Additionally, the application of radiotherapy can significantly alter the tumor microenvironment, a process mediated by the mechanisms of ionizing radiation release. This leads to the release of tumor-associated antigens and immune-stimulating factors previously concealed within tumor cells. The release of these molecules not only remains localized to the tumor but also has a systemic impact, eliciting a “distant effect” by stimulating a systemic anti-tumor immune response (28, 29). Furthermore, tumor-related vascular endothelial cells undergo changes after radiotherapy, producing more chemotactic factors. This leads to the entry of CD8+ T cells and other anti-tumor effector cells into the tumor microenvironment, demonstrating potent anti-tumor effects (30). Anti-HER-2 treatment plus radiotherapy can also play a significant role. By modulating the DNA damage repair mechanisms induced by radiation, anti-HER-2 targeted therapy helps reverse the resistance of HER-2 overexpressing cell lines to radiotherapy, thereby enhancing treatment efficacy (31). The combined application of radiotherapy and anti-HER-2 targeted therapy demonstrates a synergistic effect, providing broader possibilities and hopes for improving anti-tumor effects in patients (32, 33).

Currently, various treatment options for breast cancer are actively being explored (34). In the KAITLIN study, Ian E. et al. (35) conducted a study on HER2-positive breast cancer patients, aiming to compare the efficacy of trastuzumab with or without paclitaxel after anthracycline-based chemotherapy. The results confirmed that the three-drug regimen group had a three-year invasive disease-free survival (IDFS) of 94.2% (95% CI, 92.7-95.8), which was similar to the two-drug regimen group with a three-year IDFS of 93.1% (95% CI, 91.4-94.7). Bartels et al. (36) compared axillary lymph node dissection with axillary radiotherapy for cT1-2, lymph node-negative breast cancer patients with positive sentinel lymph node biopsy. The study found that the ten-year axillary recurrence rate (1.82% vs. 0.93%) was similar between the radiotherapy group (n=681) and the surgery group (n=744), and no differences were observed in OS (HR, 1.17) and PFS (HR, 1.19) between the two groups. In the study by Strnad et al. (37), postoperative accelerated partial breast irradiation (APBI) was compared with WBRT for early-stage breast cancer patients. The ten-year local recurrence rate was 1.58% (0.37-2.8) for the WBRT group and 3.51% (1.99-5.03) for the APBI group, with a difference of 1.93% (95% CI 0.018-3.87; p=0.074) in ten-year incidence rates. The APBI group also had a lower rate of grade 3 late adverse events compared to the WBRT group (P=0.021). In a phase III controlled study conducted by Xu et al. (38), the combination of darolutamide and fluvestrant demonstrated superior progression-free survival (PFS) (15.7 months, 95% CI 11.1-not reached) compared to placebo plus fluvestrant (7.2 months, 95% CI 5.6-9.2) for breast cancer patients.

In our study, both before and after matching, the RT group showed longer survival benefits compared to non-RT group (51.7 vs. 33.9, P<0.0001; 57.2 vs. 34.1, P<0.0001). These ongoing explorations in breast cancer treatment allow for a deeper understanding of the pathological mechanisms and biological characteristics of breast cancer, leading to more personalized treatment options. In this continuously evolving field, different treatment choices and techniques are emerging, providing more hope and opportunities for breast cancer patients.

Furthermore, to further clarify the impact of palliative radiotherapy on the prognosis of metastatic breast cancer, we conducted subgroup analyses based on different sites of metastasis. In the combined subgroup analyses involving bone, brain, liver, and lung metastasis, the RT group still demonstrated longer mOS compared to non-RT group. Pérez-Montero et al. (24) conducted a study to investigate the efficacy of SBRT in breast cancer patients with bone metastasis, reporting a 2-year local control rate and OS rate of 86.6% and 90.6%, respectively. Oymak et al. (39) studied 58 breast cancer patients with liver metastasis who received SBRT between April 2013 and March 2021. In this study, the 2-year OS, PFS, and local control rates were 71.4%, 27.5%, and 86.8%, respectively. Franceschini et al. (40) applied SBRT to breast cancer patients with lung or liver metastasis, and all patients tolerated the treatment well without experiencing grade 3/4 toxicity. Currently, there is limited reporting on radiotherapy for distant metastasis in breast cancer, but these studies also demonstrate that radiotherapy is an effective and safe treatment modality for breast cancer distant metastasis.

Of course, our study still has some limitations (41, 42). Firstly, our study involved patients who used different types of targeted drugs and chemotherapy drugs, and this heterogeneity in treatment medications may impact our study results. Secondly, despite using PSM to eliminate baseline errors, the potential heterogeneity of patients remains uneliminated. In addition, our study primarily focused on the analysis of overall survival, lacking a comprehensive discussion of treatment safety and progression-free survival, among other important indicators. Larger prospective studies will provide the medical community with more objective, authentic, and meaningful treatment evidence to improve treatment responses and prolong survival in patients.

## 5 Conclusion

For HER-2 positive metastatic breast cancer patients, those who receive radiotherapy experience better survival benefits compared to those who do not receive radiotherapy. These meaningful results can better assist clinicians in decision-making.

## Data availability statement

The original contributions presented in the study are included in the article/supplementary material. Further inquiries can be directed to the corresponding author.

## Ethics statement

This retrospective study was approved by the Ethics Committee of West China Hospital and complied with the standards of the Declaration of Helsinki. The Ethics Committee abandoned the informed consent form because it was a retrospective study. The studies were conducted in accordance with the local legislation and institutional requirements. Written informed consent for participation was not required from the participants or the participants’ legal guardians/next of kin because this retrospective study was approved by the Ethics Committee of West China Hospital and complied with the standards of the Declaration of Helsinki. The Ethics Committee abandoned the informed consent form because it was a retrospective study.

## Author contributions

XTL: Data curation, Supervision, Validation, Writing – original draft. XZ: Writing – original draft, Data curation, Formal analysis. HX: Formal analysis, Supervision, Validation, Writing – original draft. JW: Data curation, Supervision, Validation, Writing – original draft. XGL: Data curation, Supervision, Validation, Writing – original draft. YW: Data curation, Writing – original draft. LH: Data curation, Writing – original draft. JM: Data curation, Writing – original draft. GL: Data curation, Writing – original draft. LL: Data curation, Supervision, Writing – original draft.
